# Complete Genome Sequence of *Clostridium* sp. Strain DL-VIII, a Novel Solventogenic *Clostridium* Species Isolated from Anaerobic Sludge

**DOI:** 10.1128/genomeA.00605-13

**Published:** 2013-08-08

**Authors:** Safiyh Taghavi, Javier A. Izquierdo, Daniel van der Lelie

**Affiliations:** Center for Agricultural and Environmental Biosolutions, RTI International, Research Triangle Park, North Carolina, USAa; Biosciences Department, Brookhaven National Laboratory, Upton, New York, USAb

## Abstract

We report the genome sequence of *Clostridium* sp. strain DL-VIII, a novel Gram-positive, endospore-forming, solventogenic bacterium isolated from activated anaerobic sludge of a wastewater treatment plant. Aside from a complete *sol* operon, the 6,477,357-bp genome of DL-VIII reveals genes for several unique enzymes with applications in lignocellulose degradation, including two phenolic acid decarboxylases.

## GENOME ANNOUNCEMENT

Growing demand for alternative liquid fuels has sparked renewed interest in butanol-producing clostridia such as *Clostridium acetobutylicum* and *Clostridium beijerinckii*, but such model organisms that perform acetone-butanol-ethanol (ABE) fermentations are limited in number (1). Here we describe the genome sequencing of a novel solventogenic *Clostridium* species isolated from anaerobic sludge, *Clostridium* sp. strain DL-VIII.

*Clostridium* sp. DL-VIII genomic DNA was sequenced using a combination of Illumina (2) and 454 technologies (3). The initial draft assembly contained 89 contigs in one scaffold. The 454 Titanium and 454 paired-end data were assembled with Newbler version 2.3. Newbler consensus sequences were computationally shredded into 2-kb overlapping shreds. Illumina sequencing data were assembled with Velvet, version 1.0.13 (4), and the consensus sequence was computationally shredded into 1.5-kb overlapping shreds. All shreds were integrated with the 454 library read pairs using parallel Phrap, version SPS 4.24 (High Performance Software, LLC). Consed (5–7) was used in the finishing process. Illumina data were used to correct potential base errors and increase consensus quality using Polisher (Alla Lapidus, unpublished). Possible misassemblies were corrected using gapResolution (C. Han, unpublished), or Dupfinisher (8) or through subcloning. Gaps between contigs were closed in Consed, by PCR and Bubble PCR primer walks. The final assembly is based on 245.2 Mb of 454 draft data, providing an average 38.6× coverage, and 685.3 Mb of Illumina draft data, providing an average 107.8× coverage of the genome.

The complete genome is composed of a circular chromosome of 6,477,357 bp (30.1% GC content), including 6,230 coding genes, 79 tRNAs, and 13 rRNA operons. A total of 5,138 coding genes (82.5% of the total) have putative functions assigned on the basis of annotation. BLAST results of the 16 rRNA gene operons point to *C. beijerinckii* NCIMB 8052 as the closest relative, with 98.7% to 99.5% sequence identity. However, there are 1,483 genes in DL-VIII (23.8% of the total) not present in *C. beijerinckii*, while the remaining 4747 shared genes between *C. beijerinckii* and DL-VIII have an average amino acid sequence similarity of 78.42%. Therefore, *Clostridium* sp. DL-VIII represents a novel solventogenic species.

Among the unique genes found in strain DL-VIII are the first two phenolic acid decarboxylases detected in a *Clostridium* species (CDLVIII_0111 and CDLVIII_2616), which may play a role in lignin depolymerization. Likewise, 19 glycosyl hydrolases from 9 families were not found in *C. beijerinckii*, with particular prominence of hemicellulose-specific enzymes from families GH10, GH39, and GH43. An analysis of the *sol* operon in this strain reveals distinct sequence differences with closely related solventogenic clostridia, such as *C. beijerinckii*, *C. saccharobutylicum*, and *C. saccharoperbutylacetonicum*, in key steps catalyzed by butyraldehyde dehydrogenase (83.8 to 85.5% sequence identity), 3-oxoacid CoA-transferase (79.6 to 85.9% identity) and acetoacetate dehydrogenase (91.4 to 92.6% identity).

The genome sequence of *Clostridium* sp. DL-VIII will contribute to our knowledge not only of the limited diversity of solventogenic clostridia but also of other processes relevant to biofuels production, such as lignin depolymerization.

### Nucleotide sequence accession number.

The complete genome sequence of *Clostridium* sp. DL-VIII is deposited in GenBank under accession number CM001240.

## References

[1] Papoutsakis ET 2008 Engineering solventogenic clostridia. Curr. Opin. Biotechnol. 19:420–429 18760360 10.1016/j.copbio.2008.08.003

[2] Bennett S 2004 Solexa Ltd. Pharmacogenomics 5:433–438 15165179 10.1517/14622416.5.4.433

[3] Margulies M Egholm M Altman WE Attiya S Bader JS Bemben LA Berka J Braverman MS Chen YJ Chen Z Dewell SB Du L Fierro JM Gomes XV Godwin BC He W Helgesen S Ho CH Irzyk GP Jando SC Alenquer ML Jarvie TP Jirage KB Kim JB Knight JR Lanza JR Leamon JH Lefkowitz SM Lei M Li J Lohman KL Lu H Makhijani VB McDade KE McKenna MP Myers EW Nickerson E Nobile JR Plant R Puc BP Ronan MT Roth GT Sarkis GJ Simons JF Simpson JW Srinivasan M Tartaro KR Tomasz A Vogt KA Volkmer GA Wang SH Wang Y Weiner MP Yu P Begley RF Rothberg JM 2005 Genome sequencing in microfabricated high-density picolitre reactors. Nature 437:376–380 16056220 10.1038/nature03959 PMC1464427

[4] Zerbino DR Birney E 2008 Velvet: algorithms for de novo short read assembly using de Bruijn graphs. Genome Res. 18:821–829 18349386 10.1101/gr.074492.107 PMC2336801

[5] Ewing B Green P 1998 Base-calling of automated sequencer traces using Phred. II. Error probabilities. Genome Res. 8:186–194 9521922

[6] Ewing B Hillier L Wendl MC Green P 1998 Base-calling of automated sequencer traces using Phred. I. Accuracy assessment. Genome Res. 8:175–185 9521921 10.1101/gr.8.3.175

[7] Gordon D Abajian C Green P 1998 Consed: a graphical tool for sequence finishing. Genome Res. 8:195–202 9521923 10.1101/gr.8.3.195

[8] Han C Chain P 2006 Finishing repeat regions automatically with Dupfinisher, p 141–146 In Arabnia HR Valafar H (ed), Proceedings of the 2006 International Conference on Bioinformatics & Computational Biology. CSREA Press, Las Vegas, NV.

